# Neurophysiological, balance and motion evidence in adolescent idiopathic scoliosis: A systematic review

**DOI:** 10.1371/journal.pone.0303086

**Published:** 2024-05-22

**Authors:** Matilde Paramento, Edoardo Passarotto, Maria Chiara Maccarone, Michela Agostini, Paola Contessa, Maria Rubega, Emanuela Formaggio, Stefano Masiero

**Affiliations:** 1 Department of Neurosciences, Section of Rehabilitation, University of Padova, Padova, Italy; 2 Department of Information Engineering, University of Padova, Padova, Italy; 3 Padova Neuroscience Center, University of Padova, Padova, Italy; 4 Orthopedic Rehabilitation Unit, Padova University Hospital, Padova, Italy; 5 Ospedale Riabilitativo di Alta Specializzazione di Motta di Livenza, Motta di Livenza, Treviso, Italy; Iran University of Medical Sciences, IRAN, ISLAMIC REPUBLIC OF

## Abstract

**Background:**

Adolescent idiopathic scoliosis (AIS) is a spinal deformity that affects approximately 4% of the world’s population. Several hypotheses regarding the etiology of AIS have been investigated. In the last decades, impaired visual-spatial perception, alterations in spatial body orientation and sensory integration deficits have been documented.

**Objective:**

We aimed to summarize the neurophysiological, balance, and motion evidence related to AIS published in the last fifteen years, between January 2008 and April 2023. Both observational and interventional studies were considered. Only studies using quantitative assessment methods, such as electroencephalography (EEG), electromyography (EMG), magnetic resonance imaging (MRI), somatosensory evoked potentials, force platform, or motion capture, were included.

**Methods:**

1250 eligible records identified from online database searching were filtered by duplicate removal, title and abstract screening, and qualitative analysis. 61 articles met the inclusion criteria (i.e., Cobb range 10°-35°, age range 10-18 years) and were summarized.

**Results:**

We found significant evidence of impaired standing balance in individuals with AIS who greatly rely on visual and proprioceptive information to stay upright. EMG studies frequently reported an increased activity on the convex side of the intrinsic spinae muscles. EEG data show increased delta and theta power, higher alpha peak frequencies, and significant suppression in the alpha and beta bands in subjects with AIS during standing tasks. MRI studies report changes in white matter structures, differences in the vestibular system, and abnormal cortical activations over motor-related areas in subjects with AIS. Bracing appears to be an effective treatment for AIS, leading to improvements in static balance and gait. Methodological issues prevent reliable conclusions about the effects of other treatment options.

**Conclusions:**

This review underscores the importance of quantitative assessment methods to explore the etiology and pathophysiology of AIS. Further research is needed to measure the impact of physical therapy and orthotic treatments on the neurophysiological mechanisms of the disease.

## Introduction

Adolescent idiopathic scoliosis (AIS) is a spinal deformity characterized by the presence of a frontal plane curve, with a Cobb angle greater than 10 degrees, axial rotation, and a reduction of sagittal curves, manifested between 10 and 18 years of age [1, 2]. Epidemiological studies suggest that the incidence of AIS ranges from 0.14% to 0.43%, with a prevalence estimated at approximately 4% of the global school population [3, 4]. Currently, the topographic classification of AIS includes five main categories: cervical, cervicothoracic, thoracic, thoracolumbar, and lumbar. The severity of AIS is categorized based on the Cobb angle degrees: low (up to 20 degrees), moderate (21–35 degrees), moderate to severe (36–40 degrees), severe (41–50 degrees), severe to very severe (51–55 degrees), and very severe (56 degrees or more) [2]. Significantly, there is a notable gender disparity, with girls having a 3.6:1 risk of severe progression [3, 4]. Progression of AIS, if left untreated, can result in severe trunk deformities that affect thoracic capacity, biomechanics, exercise capacity, and general fitness, all of which impact the quality of life. Approaches to treating AIS range from conservative management to the use of braces or surgical intervention. Due to its effects on physical, psychological, biomechanical, neuromotor, and cardiorespiratory functions in young individuals, AIS has attracted considerable interest [5, 6]. Despite the growing interest in AIS within the scientific community, a comprehensive understanding of its causes and pathophysiology remains unclear. Several hypotheses for the etiology of AIS have been investigated, including potential defects in the central nervous system (CNS) control of body posture, anomalous interactions among hormones crucial for growth processes (melatonin, growth hormone), altered body schema, genetic determinants of cell membrane defects associated with collagen, and abnormalities in skeletal muscles [7–11]. Additionally, biomechanical alterations in the spine appear to contribute to the complex array of factors influencing the development and progression of AIS [12]. There is increasing evidence for cortical involvement in AIS: impairments in visuospatial perception, alterations in body spatial orientation, and sensory integration disorders have all been documented [13, 14]. Furthermore, according to the neurodevelopmental theories of AIS, a temporal imbalance between musculoskeletal maturation and central sensorimotor integration processes in the posterior parietal cortex could lead to improper trunk muscle responses and initiate the scoliosis process [15]. Therefore, the assessment of subjects with AIS is beginning to include not only physical, structural, and biomechanical evaluations, but also neurophysiological approaches, as well as gait and balance assessments [4]. Because they are noninvasive and relatively low cost, surface electromyographic (EMG) and electroencephalographic (EEG) data could be a useful tool for investigating the neurophysiology of AIS. EMG data combined with motion data allow the detection of movement impairments and possible compensatory movement strategies due to motor control abnormalities. Motion data are often coupled with force platforms, which aim to quantify balance and gait by measuring the ground reaction forces generated by a body standing on or moving over them.

EEG and functional magnetic resonance imaging (fMRI) data allow detection of motor planning and intention. Findings from fMRI and EEG have revealed abnormal activation in the sensorimotor brain network during simple motor tasks and alterations in cortical processing related to balance control [16, 17]. Several studies are also investigating muscle activity, gait patterns, and balance using reflective markers for motion capture, EMG sensors and force platforms in subjects with AIS. [18–20]. Abnormal somatosensory evoked potentials (SEPs), i.e., electrical signals recorded from the nervous system in response to stimulation of peripheral nerves, have also been found in subjects with AIS in relation to impaired static balance functionality [21]. However, to date, no literature has synthesized the results of neurophysiological, gait and balance studies in subjects with AIS and has determined the quality of these studies. Knowledge in this field can contribute to the development of diagnostic and treatment protocols that take into account the complex etiology of AIS. It may also help researchers to identify the limitations of the current literature and improve the design of their studies. The aim of this review is to summarize the evidence in the literature and report whether early-stage neurophysiological, balance and gait changes can be identified in this specific population.

## Materials and methods

The review was conducted by three reviewers, EP, MP, and MCM, from different scientific disciplines (neuroscience, biomedical engineering, and physical medicine and rehabilitation) whose expertise ensured a comprehensive consideration of all aspects of the study. The research question, jointly formulated by the authors, was defined as: “What is the evidence for the role of neurophysiological methods in identifying early-stage functional and neurostructural alterations in individuals diagnosed with AIS?”. The PICOS framework was applied (Population: subjects diagnosed with AIS between the age of 10 and 18 years; Intervention: quantitative neurophysiological measures; Comparison: none/healthy subjects; Outcome: identification of functional and neurostructural alterations; Study Design: clinical trials).

### Inclusion criteria

The analysis included original research articles published from January 2008 up to July 2023, with repetitive or unrelated studies excluded. This allowed us to compare evidence that was collected with similar assessment devices. In fact, advancements in behavioral, electrophysiological, and neuroimaging assessment methods may make recent evidence not comparable to older evidence. Case reports, reviews, commentaries, letters to the editor, methodological and conference papers, and studies published before January 2008 were excluded. Participants were required to have a mean age ranging from 10 to 18 years. All subjects included in the review had a radiographic diagnosis of AIS by an expert clinician. All AIS subtypes, such as single, double, left, and right curvature, were accepted. Subjects with secondary scoliosis, congenital abnormalities, vertebral scoliosis attributable to syndromic conditions, and other comorbidities were excluded. Since neurophysiological methods are used in the intraoperative assessment of subjects with AIS, but they are not commonly used during clinical evaluations of the onset and progression of scoliosis, this review focused on individuals with mild to moderate AIS who were not candidates for surgery. As the review mainly focused on subjects with mild and moderate scoliosis, studies were required to report a mean Cobb angle between 10 and 35 degrees and no history of corrective surgery. All studies that used quantitative neurophysiological assessment methods, such as EEG, EMG, MRI, SEPs, force platform, or motion capture systems, to identify functional and neurostructural abnormalities in subjects with AIS were eligible for inclusion in the review. These included cross-sectional and longitudinal evidence from single-group studies and studies that included control groups of comparable healthy subjects (i.e. age- and sex-matched). On the contrary, studies that focused solely on structural assessment of the spine were considered irrelevant to our purpose. Only studies available in English were included in the analyses.

### Database research

**Fig 1:**
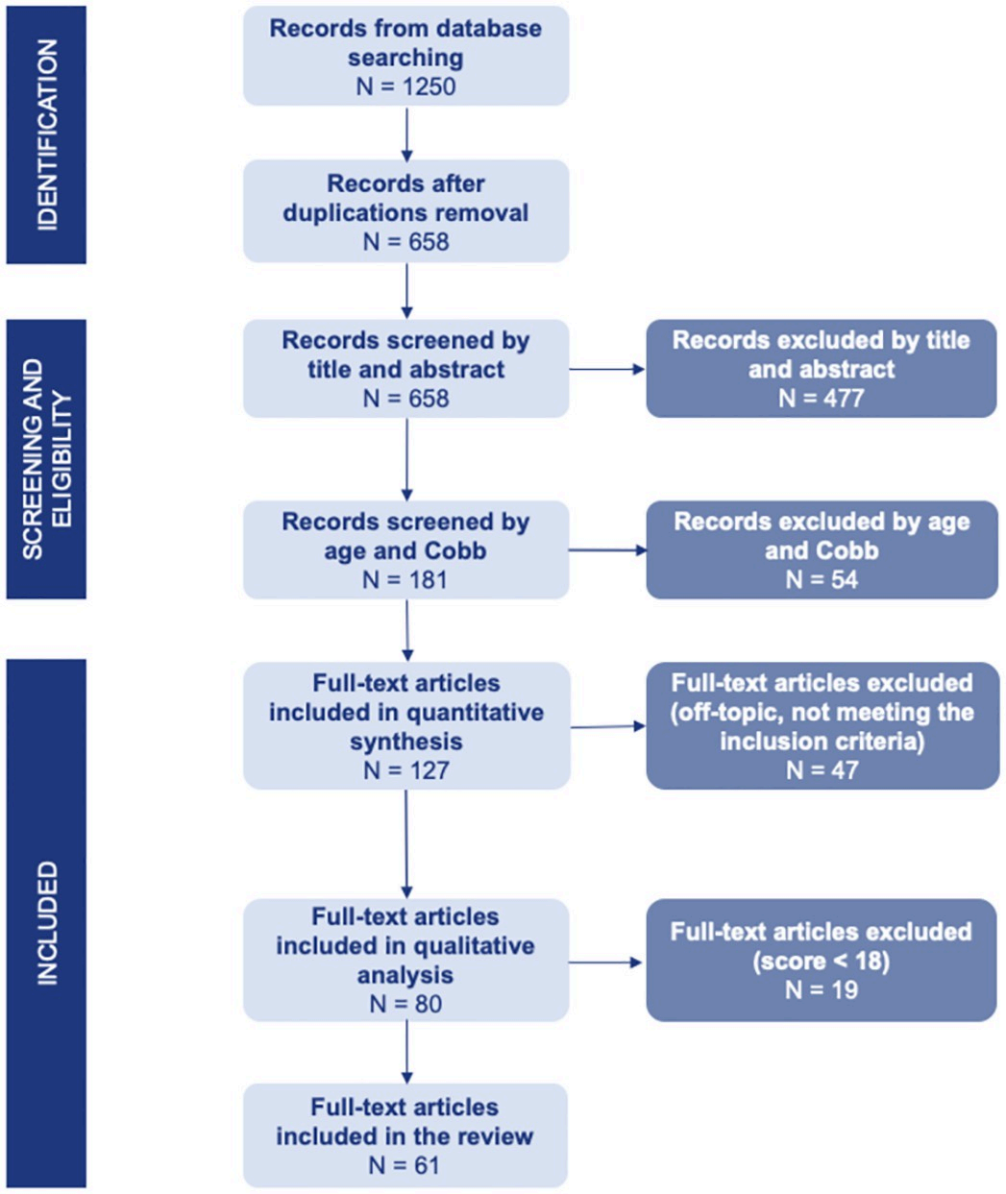
PRISMA-flow of the articles selection process.

The research was conducted using multiple search engines, including Web of Science, Pubmed, Scopus, IEEE, and Cochrane Library. The search strings were constructed as follows: the keywords for the target population were combined using the Boolean operator AND, which represents the algebraic intersection, to the keywords for each assessment tool, such as EEG, EMG, MRI, SEPs, force platform, motion capture system, magnetoencephalography (MEG), and near infrared spectroscopy (NIRS), combined using the OR Boolean operator, which corresponds to the algebraic union. We discarded studies that included pre-surgical subjects or individuals with a history of surgical treatment by exploiting the Boolean operator NOT (the strings used in the research are reported in the S1 Table). The initial database consisted of 1250 studies published between January 2008 and April 2023, identified according to the Preferred Reporting Items for Systematic Review and Meta-Analysis (PRISMA statement [22]). The Zotero software was used to eliminate duplicates. 658 articles progressed to the subsequent screening phases. The screening process consisted of two phases. First, the titles and abstracts of the selected articles were examined. Those that were considered off-topic or did not meet the inclusion criteria were not advanced to the subsequent screening phase. In this step, (1) articles that only included radiological or MRI spine assessment, (2) case reports, reviews, commentaries, letters to editor, methodologic and conference papers, and (3) articles that did not have a Digital Object Identifier (DOI) were removed from the database. 181 articles proceeded to the second screening phase. The age and Cobb angle values of the subjects included in each study were then investigated to determine if they met the inclusion criteria. Studies for which the full English text was not available were excluded from the database. 127 studies remained for full-text analysis. The two screening phases were performed by the three reviewers at the same time. If there was no unanimous agreement among the reviewers (i.e., at least one reviewer was uncertain whether to retain the article), the article proceeded to the next step. The pipeline of the database search is shown in Fig 1.

### Articles selection

The reviewers utilised a standardised form to extract pertinent information from the eligible studies. They then independently evaluated the quality of the studies based on a quality assessment inventory consisting of fourteen criteria. This quality assessment inventory has often been used to evaluate and summarize electrophysiological and orthopedic evidence from the literature [23–25].

#### Data extraction form

For each study the following information was recorded: (1) authors, year and journal of publication, (2) demographic and clinical data of the subjects, (3) study design, (4) assessment tools, (5) signal analysis pipeline, and (6) outcomes. Each of the three reviewers performed the information extraction process on a third of the eligible studies. The work of each reviewer was cross-checked by the others. A model of the data extraction form is provided in S2 Table. 47 full-text articles were excluded because they were considered off-topic or did not meet the inclusion criteria.

#### Quality assessment

For each eligible study, the reviewers evaluated its quality based on fourteen criteria adapted from the literature [23–25], grouped into seven sections (see S3 Table). These included the 1) scientific relevance and the clarity of the description of the research question, 2) inclusion and exclusion criteria, 3) data collection and processing, 4) data loss, 5) statistical approach, 6) outcomes, and 7) presentation of the results. Each reviewer assigned a score ranging from 0 to 2 for each criterion, taking into account whether the objectives were not met (0), partially met (1) or fully met (2). The scores for each specific criterion were summed and averaged among the reviewers. Therefore, each study received a score on a scale from 0 to 28. If the final score exceeded 60% of the maximum score (i.e., > 17), the article was included in the review. 80 full-text articles were included in the qualitative analysis. 61 studies met this criterion. Intraclass correlation (ICC) coefficients were computed to evaluate the agreement between the ratings assigned by the three reviewers. The results indicated an acceptable level of interrater reliability, with ICC3 = 0.76.

## Results

**Fig 2:**
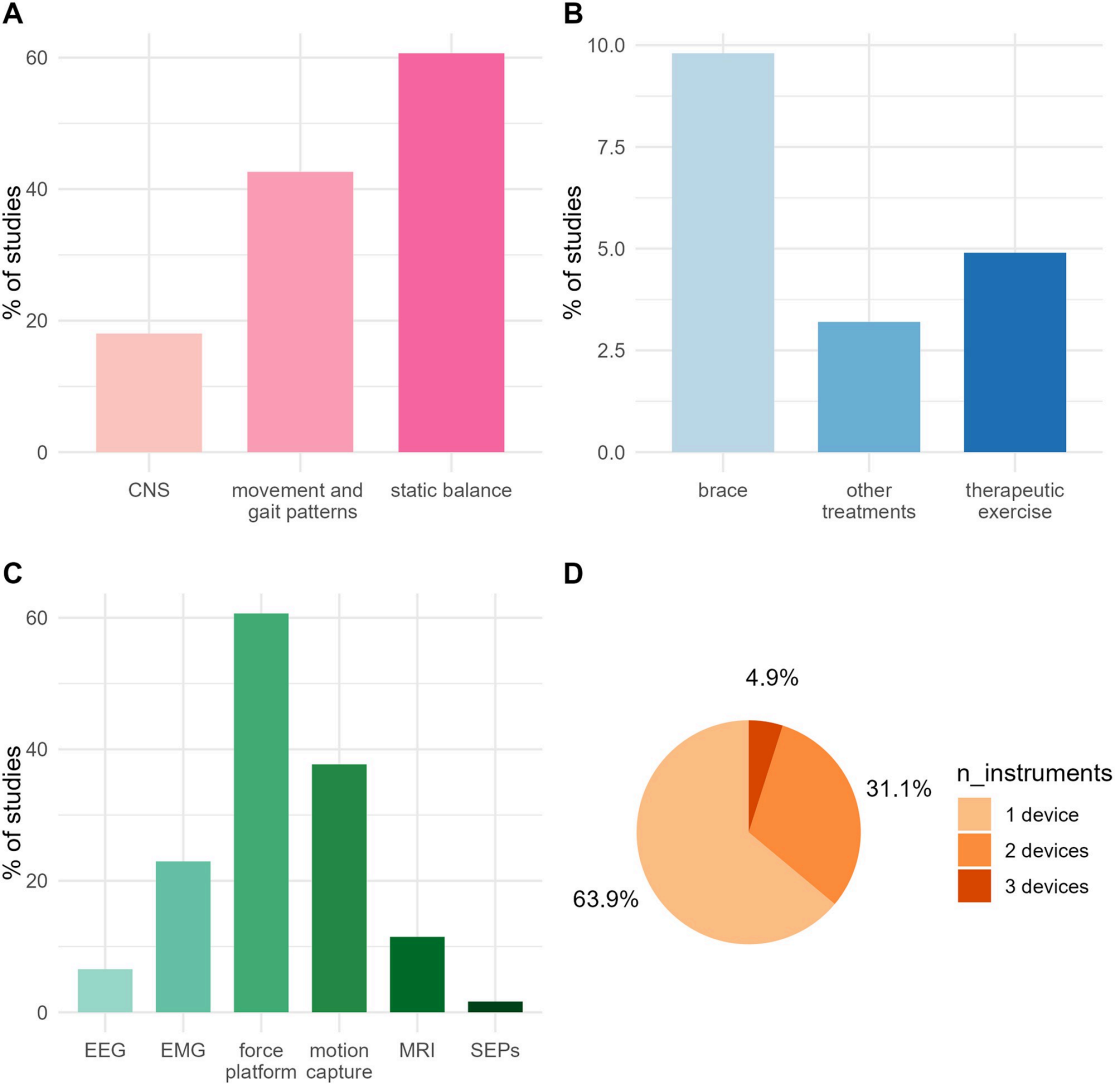
Frequency of study designs and measurement types. The selected studies (N = 61) are divided by (A) research topic, (B) type of intervention, (C) type and (D) number of assessment devices used.

61 studies were selected for the review, of which 60.6% (N = 37) used static balance assessment methods and 42.6% (N = 26) investigated movement and gait patterns. EMG measurements were used in 22.9% (N = 14) of cases, MRI in 11.5% (N = 7), EEG in 6.5% (N = 4), and SEPs in 1.6% (N = 1). 18.0% (N = 11) of the studies investigated the effects of specific treatments on the progression of the disease, including brace intervention (9.8%, N = 6), therapeutic exercise (4.9%, N = 3), and other treatments (3.2%, N = 2) such as personalized insoles and sEMG biofeedback. 9.8% (N = 6) of them implemented longitudinal study designs and 90.2% (N = 55) included control groups. More information on the frequency of study designs and measurement types is reported in Fig 2. In total, the selected studies investigated AIS in 1693 subjects, of which 92.2% (N = 1562) were females. 63.9% (N = 39) of the studies investigated AIS only in female subjects. In the remaining studies, males represented 17.6% (N = 131) of the overall sample. The mean age of the samples was 14.0 years (SD = 1.2, range = 11.7–18.9).

The quality assessment identified common risks of bias, particularly in the selection of subjects, insufficient details in the description of data processing and presentation of results, which could affect the assessment of the adequacy of the analysis. Issues related to the lack of randomization, control groups, blinding, selective reporting, and other methodological concerns were also identified. The methodological quality scores of the studies ranged between 12 and 28. Nine studies had scores greater than 25 and reported high quality evidence, while lower scores were associated with a higher risk of bias. In the result section, the reviewers grouped the studies that examined similar functional and neurostructural alterations in subjects with AIS.

### Synthesis of results on static balance

Several studies investigated deficits in postural control due to AIS during natural standing using force platforms. Generally, the center of pressure (COP) was shown to have a greater anteroposterior and mediolateral displacement [26–28], a greater sway speed [26, 28–30], and a greater sway area [27, 29, 31], and to cover a longer range [28–30] in subjects with AIS compared to healthy controls. Leteneur et al. [32] used the Morlet wavelet transform to analyze coherence between the mediolateral, anterolateral COP excursion and the corresponding vertical forces (Tz). During quiet standing, in subjects with AIS the coherence between the mediolateral COP excursion and Tz was higher in the low frequency band (0.16–0.50 Hz) and lower in the high frequency band (0.50–8.00 Hz) than in controls. Subjects with AIS were also characterized by a more posteriorly oriented mean position of the COP than healthy individuals [27, 28, 32, 33]. However, this might not be true for all subjects with AIS. For example, Catan et al. [29] showed a more anterior and leftward-oriented COP mean position in subjects with S-shaped moderate scoliosis, having right convex thoracic and left convex lumbar curves. Interestingly, Leteneur et al. [30] analyzed balance control in AIS, distinguishing between subjects with a backward trunk lean and subjects with a forward trunk lean. Subjects with AIS with a backward trunk lean showed greater anteroposterior displacement and speed of the COP compared to controls with a similar trunk lean. However, no significant differences were found between subjects with AIS and healthy controls with a forward trunk lean. Moreover, the authors also found a significant correlation between the AIS subjects’ trunk inclination and several standing balance descriptors (i.e., anteroposterior COP mean position, mediolateral COP range, anteroposterior and mediolateral COP speed). Interestingly, Kavyani et al. [34] found no significant difference in postural balance between healthy controls and subjects with AIS.

The effect of vision on postural control was investigated by having subjects stand on a force platform with either their eyes open or closed. Instead, proprioceptive information was investigated by having subjects stand on a foam mat [35–38], by means of slow oscillations of the standing platform [39], by using a movable balance platform that allowed up to 20 degrees surface tilt [38, 40] or by ankle tendons vibrations [41]. The results showed a greater reduction in postural control in subjects with AIS than in controls due to a lack of visual information [35–37, 42] and when proprioceptive information was altered by having subjects stand on a foam mat [35, 37]. The COP of subjects with AIS showed a greater sway radius, path length, path range, speed and sample entropy during demanding conditions (i.e., standing on a foam surface with eyes closed) compared to healthy controls. Furthermore, Dabrowska et al. [42] showed that postural stability was negatively related to the degree of spine rotation. Some studies found no significant difference between subjects with AIS and controls related to sensory information processing [17, 39, 41]. Paradoxically, Kuo et al. [38, 40] found significantly lower mediolateral and anteroposterior tilt angles in subjects with AIS compared to controls in all experimental conditions, including visual and somatosensory deprivation. However, they measured standing posture using a movable balance platform rather than a common firm force platform which may explain the difference in their results. Sim et al. [43] investigated the effect of vision on postural control in AIS by using an interesting analytical method. Using the discrete wavelet transform, they decomposed the signal recorded by the force platform into closed-loop (< 1.0Hz) and open-loop (> 1.0Hz) signals. The closed-loop signal was further divided into three main components, each representing a sensory system: somatosensory (0.5–1.0 Hz), vestibular (0.1–0.5 Hz), and visual (< 0.1 Hz) systems (for more information, see Sim et al. [43]). The difference in energy content between eyes-opened and eyes-closed conditions was significantly greater in subjects with AIS than in controls at frequency levels corresponding to the vestibular and somatosensory systems, suggesting a greater dependence on sensory information in subjects with AIS. Three studies by Pialasse et al. [44–46] investigated the effect of vestibular information on postural stability using bilateral galvanic vestibular stimulation (GVS) applied to the mastoid processes. Subjects were asked to stand upright on a force platform with their eyes closed. During and immediately after GVS, Pialasse et al. [44, 45] found greater body sway in subjects with AIS compared to healthy controls. These effects were not related to the degree of spine deformation. Interestingly, Pialasse et al. [46] found significant differences within subjects with AIS, with only 42.5% of the subjects showing abnormal sensorimotor control and increased response to GVS.

Three studies [34, 37, 47] investigated the effect of scoliosis treatments on balance control. Pavone et al. and Kavyani et al. [34, 47] investigated the short-term effects of wearing a brace on postural control in a quiet standing position. The results showed that braces did not improve balance in subjects with AIS, but rather increased the sway speed of the COP [47]. Kavyani et al. [34] showed that wearing a brace was only effective in reducing the mediolateral sway of the COP while standing upright on a force platform. Additionally, Khanal et al. [37] reported that after four months of wearing a brace, subjects with AIS showed a decrease in the mediolateral COP sway, while the anteroposterior COP sway and its path length significantly increased. Wang et al. [48] compared the effects of personalized insoles on AIS progression and postural balance, as a possible alternative to physical therapy. After 6 months of bracing treatment together with one of the two alternative treatments, the authors found similar improvements in terms of Cobb angle, trunk rotation, and balance control in subjects who used personalized insoles and who received physical therapy. However, their study design does not allow us to separate the effect of the two alternative treatments from the effect of bracing. Three studies investigated the effect of corrective exercises on sitting [49] and standing balance [50, 51] in subjects with AIS. After three weeks of training based on lumbar stabilization exercises, Shin et al. [49] showed significant improvements in sitting balance in both eyes-opened and eyes-closed conditions. Marin et al. [50, 51] investigated the effect of self-elongation and self-correction exercises on postural control during quiet standing. The results did not show any significant effect of self-elongation exercises on postural stability. Instead, self-correction exercises were effective in improving most balance stability descriptors, including mediolateral sway, sway area and eccentricity of the ellipse of the COP. None of these studies included control groups, which limits the reliability of their findings [49–51].

### Synthesis of results on movement and gait

Several studies investigated abnormalities in gait patterns due to AIS using motion capture systems and force platforms. Subjects with AIS showed significantly reduced stance [52, 53] and increased swing phases [52, 54] compared to controls. Abnormalities in ground reaction forces (GRF) during gait were also reported: Bruynell et al. [55, 56] found stronger anteroposterior and vertical as well as weaker mediolateral impulses of the ground reaction forces during gait initiation in subjects with AIS, while Sung et al. [57] found a greater mediolateral peak force in the dominant limb. Zhu et al. [54] showed that the ratio of impulses between the big toe and the first metatarsal was greater in subjects with AIS than in healthy subjects. Kinematic data of the lower limbs during self-paced walking suggested that AIS is characterized by reduced range of motion (ROM) of the pelvis, hip, knee, and shoulder [53, 58, 59]. Park et al. [58] showed greater in-phase and lower anti-phase coordination between the thorax and the pelvis during gait. AIS did not seem to affect walking speed, stride length, and cadence of the subjects [21, 52, 60]. Subjects with AIS showed greater gait asymmetries [54, 56, 57, 60], particularly in terms of GRF, step length, stance duration, and swing phases between the right and left feet. Yang et al. [60] confirmed a generally lower correlation between left and right body segments (i.e., shank, thigh, pelvis, trunk) in the frontal and transverse planes in subjects with AIS compared to controls. These asymmetries may result in increased energy cost and decreased muscular efficiency during gait, as reported by Mahaudens et al. [61]. Three studies investigated the effect of bracing on gait patterns in AIS. When subjects wore a brace, the ROM of the trunk, knees, pelvis, and shoulders was reduced in most planes [59, 62]. In addition, left-right asymmetry of the trunk and ankles was significantly increased immediately after bracing [59]. There were no significant differences in speed, step length, cadence and stance phase duration due to bracing [62]. Mahaudens et al. [63] investigated the long term effects of bracing on gait patterns. After 6 months of bracing, the pelvic ROM in the frontal plane was significantly increased while the shoulder ROM was significantly decreased. In addition, wearing a brace increased step length and stance phase duration while decreased step cadence.

Turgur et al. [64] investigated scapular kinematics in subjects with AIS who were asked to perform bilateral, full overhead arm elevations. During the task, subjects with AIS achieved lower bilateral elevation peaks and were characterized by greater internal, downward rotation and anterior tilt of the scapula on the convex side. On the concave side, AIS was associated with greater external, downward rotation and posterior tilt of the scapula. Sung et al. [65] examined the effect of trunk rotation on postural control in subjects with AIS and healthy controls. The ROM of the first thoracic spinous (T1) process and the first lumbar spinous (L1) process were positively correlated with the Cobb angle. Moreover, the displacement of the vector connecting T1 and L1 was lower during the anti-phase (left to right) trunk rotations in subjects with AIS than in controls. In Leteneur et al. [66], subjects were asked to perform full-body swings, reaching their maximum tilt in the anterior, posterior, right and left axes. When performing mediolateral and anteroposterior swings, subjects with AIS showed greater anteroposterior and mediolateral COP deviations than controls, respectively. Similarly, Struber et al. [31] showed that during lateral bending, subjects with AIS exhibited lower ROM of the pelvis and reduced pelvis and thorax frontal displacement.

### Synthesis of results on muscle activity

Few studies conducted an electromyographic assessment of the intrinsic spinae muscles, i.e., erector spinae, paraspinal muscles, multifidus and trapezius, during exercises aimed at activating the spinal extensor muscles in adolescents with idiopathic scoliosis. Chwala et al., Park et al., and Farhapour et al. [67–69] reported a significant predominant involvement of the convex-side in the paraspinal and erector spinae muscles both in subjects with both single- and double-curve scoliosis during symmetric and asymmetric exercises compared to examination at rest. Chan et al. and Lin et al. [70, 71] showed that subjects with AIS had greater activation over the convex side than over the concave side in the erector spinae and lower trapezius, during arm elevation. On the contrary, De Oliveira et al. and Chan et al. [70, 72] found no significant differences in electromyographic amplitudes of the erector spinae muscles in the convex and concave sides of the apex region of the scoliotic curve in the thoracic and lumbar regions during trunk isometric contractions. There was also no significant difference in muscle activity when subjects with AIS were compared with healthy controls. Farhapour et al. [73], and Kuo et al. [38, 40] recorded the activity of the intrinsic spinae muscles, i.e., erector spinae and multifidus, and leg muscles, i.e., biceps femoris, gastrocnemius and gluteus medii, from subjects with AIS during dynamic balance tests under different postural, visual, and sensory conditions. The activity of the right biceps femoris muscles and over the convex side in the erector spinae muscles was significantly greater in subjects with AIS than that in the healthy controls in the forward and backward dynamic perturbation test [73]. Kuo et al. [38] showed that dynamic balance control was particularly impaired under visual deprivation, with compensatory increases in lumbar multifidus and gluteus medii activities.

Mahaudens et al. [62, 63] assessed the effects of orthotic treatment on gait in female subjects with thoracolumbar and lumbar AIS. Bilateral lumbo-pelvic muscles in subjects with AIS were almost 40% more active during the gait cycle at the beginning of the therapy, compared to healthy subjects, and did not change after 6 months of orthotic treatment, except for EMG activity of the erector spinae that decreased significantly [63]. Hatzilazaridis et al., [74] investigated the bilateral axial and lower limb muscle activity evoked by bipolar, binaural GVS of randomly alternating polarity in scoliotic and healthy adolescents. Subjects with AIS showed smaller right ankle muscle responses during anode right/cathode left GVS compared with healthy controls. Cheung et al., [75] reported the effects of the sEMG biofeedback postural training program on female subjects with mild AIS. These subjects demonstrated more symmetric paraspinal muscle activity over the trapezius and lumbar erector spinae muscle pairs.

### Synthesis of results on the central nervous system

Formaggio et al., Fortin et al., and Lanthier et al. [17, 41, 76] used EEG systems to investigate brain oscillatory activity in standing position and altered sensory perception in AIS. None of the studies found significant group differences in the balance control. Formaggio et al. [17] reported a significant increase in delta and theta power bands over central cortical areas in subjects with AIS compared to controls. They showed a lateralization of the power in the alpha band when subjects stood upright with their eyes closed and arms raised at 90 degrees. Fortin et al. [41] found a significant suppression in alpha and beta EEG frequency bands in subjects with AIS when ankle proprioception was altered by means of ankle tendon vibrations. When vision was also removed, the authors found a significant increase in theta band power in subjects with AIS, consistent with Formaggio et al. [17]. Moreover, subjects with AIS showed a significant suppression of the beta and gamma power bands in the non-vision condition shortly after the proprioceptive alterations were interrupted. Lanthier et al. [76] found significantly higher alpha peak frequencies (APF) in central, frontal, parietal, and occipital regions in subjects with AIS compared to controls. When the experiment was repeated with eyes closed, subjects with AIS showed a significant decrease in APF, while the opposite trend was observed in the control group. Chang et al. [77] investigated visual event-related potentials (ERPs) in subjects with AIS, performing the modified rod-and-frame task, that measures the subjective visual verticality, in different standing positions (i.e., feet together, feet apart and tandem standing). No group differences were found in the neurophysiological data. An interesting study by Lao et al. [21] investigated the relationship between cortical SEPs and gait abnormalities in AIS. The authors measured the latency of SEPs (N37) after stimulation of the posterior tibial nerve. Eight out of the 18 subjects with AIS included in the study showed longer SEP latencies. Interestingly, only subjects with AIS with abnormal SEPs differed significantly from healthy controls in terms of ground reaction forces and joint motion asymmetry during gait.

A few studies have investigated abnormalities in the CNS by using MRI techniques. Shi et al. [78] found significant neurostructural differences in subjects with AIS compared to healthy controls. Using voxel-based morphometry, the authors identified a white matter attenuation in the genu of the corpus callosum as well as in the left internal capsule. However, these differences were only identified in subjects with a left scoliotic curve (N = 9) and did not generalize to right spinal curvatures (N = 20). Hitier et al. and Shi et al. [79, 80] identified significant differences in the morphoanatomy of the vestibular system in subjects with AIS. Using structural MRI, they found asymmetry in the inner ear. In fact, the semicircular canals on the left side showed abnormal orientation, especially the lateral semicircular canal, which was more vertically oriented in the AIS than in the control group. No difference was observed between subjects with right and left spine curvature. Domenech et al. [16] implemented functional MRI to investigate cortical activations in subjects with AIS during a simple, self-paced task, i.e., opening and closing one fist for 60 seconds at a rate of approximately 1 Hz. The results showed differences in activation patterns in motor-related cortical areas between AIS and healthy subjects, especially an increase in the number of activated voxels in the supplementary motor area in the hemisphere contralateral to the moving hand. Three studies [15, 81, 82] investigated white matter connectivity using diffusion tensor imaging (DTI) methods. Xue et al. [81] found reduced fractional anisotropy (FA) in the corpus callosum of subjects with AIS, especially in the genu and splenium areas of the left hemisphere. FA was also reduced in fibers connecting the primary somatosensory cortex with the visual cortex. Furthermore, Kong et al. [82] identified significant reductions in FA and increased mean diffusivity in the medulla oblongata and between the first and fifth cervical vertebrae (i.e., C1 and C5) in subjects with AIS compared to controls. Finally, Noriega-Gonzalez et al. [15] reported a significant increase in FA in fibers that connect motor areas and the cingulate gyrus in subjects with AIS. A summary of the result section is provided in Table 1.

**Table 1:** Summary of the results.

Research topic	Evidence found in subjects with AIS compared to healthy controls
**Static balance**	Greater anteroposterior and mediolateral displacement Greater sway speed and area Reduced postural control in condition of lack of vision and altered proprioception Decreased mediolateral sway of the COP after long-term brace treatment
**Movement and gait**	Reduced range of motion of the pelvis, hip, knee and shoulder Asymmetric ground reaction forces, step length, stance duration, and swing phases during gait
**Muscle activity**	Greater activation over the convex side in the intrinsic spinae muscles
**Central Nervous System**	Increased delta and theta power Higher alpha peak frequencies Altered white matter structure and connectivity

## Discussion

The objective of the current review is to provide a summary of the neurophysiological, balance, and gait assessment findings in subjects with AIS that have been published in the last fifteen years. In particular, the review details the use of different assessment tools to identify functional and neurostructural abnormalities in subjects with AIS, also compared with healthy controls.

Force platform and motion capture evidence indicated standing balance deficits in subjects with AIS, with variations depending on the type of the curvature and lean of the trunk [26, 27, 30]. In addition, the impact of visual and proprioceptive information alteration or deficiency on postural control appeared to be more pronounced in subjects with AIS compared to their healthy counterparts [37, 42]. These findings are consistent across various assessment tools, such as stabilometric, EMG, EEG, and MRI data, revealing abnormal processing of sensory information.

EMG assessments of intrinsic spinae muscles in AIS revealed varying findings. Some studies highlighted significant convex-side involvement [69, 71], while others reported no significant differences in electromyographic amplitudes [70]. Dynamic balance tests also showed increased EMG activity on the convex side of the erector spinae muscles [73].

In EEG studies, researchers observed increased theta and alpha power, higher alpha peak frequencies and significant suppression in the alpha and beta bands during vibration applied to the ankle in subjects with AIS during standing tasks [17, 41, 76]. Lao et al. [21] investigated the relationship between SEPs and gait patterns. They found a substantial association between longer N37 latencies, ground reaction forces and joint motion asymmetry during gait in AIS.

Through MRI, white matter changes and altered white matter connectivity were observed in the corpus callosum and left internal capsule, particularly in the left spinal curvatures [78]. Differences in the vestibular system included morphological asymmetries between the left and the right of the inner ears, particularly in the lateral semicircular canal. fMRI studies evidenced abnormal cortical activation in motor-related areas during hand movement task [79, 80].

This review provided quantitative insights into the effectiveness of AIS treatments. Bracing emerged as an effective intervention in subjects with AIS, demonstrating improvements in static balance (i.e., decrease in mediolateral COP sway) [34, 37]. Its effects were not necessarily directed toward normalization of gait and balance parameters. Indeed, the influence of treatments on trunk segment mobility appeared to yield mixed outcomes, as both increased and decreased mobility have been observed (i.e., increased pelvic and decreased shoulder ROM) [59–63]. Bracing and posture training based on EMG biofeedback demonstrated inconsistent effects on muscle activity [63, 75]. The quality of studies investigating the effects of physiotherapy and self-correction on postural control in AIS is questionable, and reliable conclusions are challenging due to design limitations, such as the absence of control groups, despite some promising results that indicate a reduction in body sway in sitting and standing positions.

The findings presented here are in line with previous reviews investigating the pathophysiology and treatment of AIS. However, we chose a rather pragmatic approach: while other works focus on specific subsets of evidence such as anatomical abnormalities or treatments (i.e., [83, 84]), our review prioritized the assessment methods. Thus, our results provide a clear, data-driven outlook that leaves less room for subjective inference.

Our review has highlighted several strengths in the included studies. Innovative research designs could make significant advances in our understanding of the pathophysiology of the disease. For instance, exploring the relationship between GVS response and balance control could help identify meaningful neurophysiological alterations in subjects with AIS. In addition, some studies have employed advanced analytical methods, such as the decomposition of the COP signal through wavelet transformation, which provide interesting information on sensory information processing during static posture.

We found a few important limitations in the literature on AIS. In the reviewed studies, research design, experimental tasks, and analytical approaches were greatly heterogeneous. On the one hand, this diversity can enhance the ecological validity and generalizability of the reported findings. On the other hand, the absence of standardized testing procedures and analytical methods makes it difficult to compare results across publications and to draw robust conclusions. Future studies should implement well-established tasks and quantitative measurements to ensure comparability of results [85]. Once these foundational criteria are met, researchers are encouraged to explore innovative designs and analytical techniques. The number of studies that examine structural and functional abnormalities of the CNS in subjects with AIS is limited. In particular, to date, no investigation has been conducted into the possible effects of AIS treatment on brain oscillatory activity or on the neurostructural abnormalities identified in the existing literature. We also observed a tendency toward permissive inclusion criteria in studies that explore these aspects. These gaps underscore the need for future research to specifically address these issues and contribute to a more comprehensive and nuanced understanding of the implications of CNS in the development and progression of AIS. Another aspect to be considered is that only 21.3% (N = 13) of the included studies involved subjects who did not undergo any corrective treatment during the investigation. This introduces an important issue, suggesting that the abnormalities reported in the literature might not be attributed only to the nature of the disease, but could potentially be influenced by the effects of the treatments administered. Finally, only a few studies conducted analyses within subjects with AIS, revealing significant within-group differences related to factors such as the side of the main curvature. Consideration of AIS subtypes could be crucial in investigating functional abnormalities.

The present review has some limitations. First, our focus was primarily on subjects with mild or moderate scoliosis, deliberately minimizing the inclusion of severe AIS cases in our analyses. Therefore, some of the abnormalities highlighted in this review may be more indicative of specific scoliosis subtypes. Second, the quality assessment inventory used in this study has not been rigorously validated, although it has been previously used in several systematic reviews in the same research area. Third, our assessment did not consider the evidence in relation to the statistical power or sample size of the respective studies. In essence, the findings of a study with a small group of subjects were assigned equal relevance to those of larger cohorts, if they met our inclusion and quality criteria. A proper meta-analysis might be necessary to investigate the magnitude of the effects reported in this review. However, we believe that the large heterogeneity in study designs and assessment methods discussed above may affect the quality and reliability of meta-analytical studies.

In conclusion, this review underscores the growing interest in neurophysiological assessments in subjects with AIS, with the exploration of neurological alterations from various perspectives. Existing evidence reveals standing balance deficits and altered postural control in subjects with AIS. Bracing treatment appears to play a role in improving static balance. However, due to the heterogeneity in the studies, there is a need for further research with standardized testing procedures to measure the impact of physical therapy and orthotic treatments on the neurophysiological mechanisms of AIS.

## Supporting information

S1 Table Search strings. (PDF)

S2 Table Data extraction form. (PDF)

S3 Table Quality assessment. (PDF)

S1 Checklist PRISMA 2020 main checklist. (PDF)
