# Supplementary material for: Neurophysiological, balance and motion evidence in adolescent idiopathic scoliosis: A systematic review
Source: PLoS One. 2024 May 22;19(5):e0303086. doi: 10.1371/journal.pone.0303086 (PMC11111046; doi:10.1371/journal.pone.0303086)
Supplement: S1 Table — (PDF) [file pone.0303086.s001.pdf]

S1 Table

Search strings.

**Search engines**	**Search strings**
**SCOPUS**	TITLE-ABS-KEY ((**idiopathic** AND **scoliosis**) AND (**adolescent** OR **adolescence**) AND (**balance** OR (**force** AND **platform**)) OR (**gait** AND **analysis**) OR **eeg** OR **emg** OR **mri** OR **fmri** OR **meg** OR **nirs** OR **sep** OR **electroencephalography** OR **electromyography** OR **neuroimaging** OR **evoked-potentials** OR (**magnetic** AND **resonance** AND **imaging**) OR **magnetoencephalography** OR (**infrared** AND **spectroscopy**) OR (**near-infrared** AND **spectroscopy**) OR (**evoked** and **potentials**) AND NOT (**operative** OR **intraoperative** OR **surgery** OR **surgical**)) AND PUBYEAR > 2007 AND PUBYEAR < 2024
**Web of Science**	TS=((**idiopathic** AND **scoliosis**) AND (**adolescent** OR **adolescence**) AND (**balance** OR (**force** AND **platform**) OR (**gait** AND **analysis**) OR **eeg** OR **emg** OR **fmri** OR **mri** OR **meg** OR **nirs** OR **sep** OR **neuroimaging** OR **electroencephalography** OR **electromyography** OR (**magnetic** AND **resonance** AND **imaging)** OR **magnetoencephalography** OR (**infrared** AND **spectroscopy)** OR (**near-infrared** AND **spectroscopy)** OR **evoked-potentials** OR (**evoked** and **potentials**)) NOT (**operative** OR **intraoperative** OR **surgery** OR **surgical**)) Note: results have been manually refined and further filtered by publication year.
**PUBMED**	(**idiopathic scoliosis** [Title/Abstract]) AND (**adolescent** [Title/Abstract] OR **adolescence** [Title/Abstract]) AND (**balance** [Title/Abstract] OR **force platform** [Title/Abstract] OR **mri** [Title/Abstract] OR **eeg** [Title/Abstract] OR **emg** [Title/Abstract] OR **mri** [Title/Abstract] OR **fmri** [Title/Abstract] OR **meg** [Title/Abstract] OR **nirs** [Title/Abstract] OR **sep** [Title/Abstract] OR **neuroimaging** [Title/Abstract] OR **electroencephalography** [Title/Abstract] OR **electromyography** [Title/Abstract] OR **magnetic resonance imaging** [Title/Abstract] OR **magnetoencephalography** [Title/Abstract] OR **near infrared spectroscopy** [Title/Abstract] OR **near-infrared spectroscopy** [Title/Abstract] OR **evoked-potentials** [Title/Abstract] OR **evoked potentials** [Title/Abstract]) NOT (**operative** [Title/Abstract] OR **intraoperative** [Title/Abstract] OR **surgery** [Title/Abstract] OR **surgical** [Title/Abstract]) AND (2008:2023[pdat])
