# Supplementary material for: Neurophysiological, balance and motion evidence in adolescent idiopathic scoliosis: A systematic review
Source: PLoS One. 2024 May 22;19(5):e0303086. doi: 10.1371/journal.pone.0303086 (PMC11111046; doi:10.1371/journal.pone.0303086)
Supplement: S2 Table — (PDF) [file pone.0303086.s002.pdf]

S2 Table

Data extraction form.

**Organizational aspects**	
**Reviewer**	date, checked by
**Article information**	list of authors, year of publication, journal/source
**Fate**	EX/IN-cluding, decision pending
**Assessment tool used**	force platform, motion capture system, EMG, EEG, MRI, SEPs, others
**Short description**	
**Aim**	
**Results in brief**	
**Study design**	
**Study design**	longitudinal (yes/no), comparative (yes/no), healthy controls (yes/no)
**Subjects**	number, age, sex
**Scoliosis parameters**	Cobb angle
**Treatment history**	therapeutic exercises, brace, surgery, not mentioned
**Intervention**	treatment, period
**Task**	conditions, number of trials, duration
**Balance and motion**	
**Force platform**	sampling frequency (fs), analyses
**Motion capture system**	fs, markers (number and anatomical location), analyses
**Other devices**	description, methods, analyses
**EMG**	
**Methods**	fs, number of channels, invasive/non invasive, muscles, filtering, normalization
**Analyses**	frequency analysis, root mean square, synergies
**EEG and SEPs**	
**Methods**	fs, number of channels, filtering
**Analyses**	frequency analysis, alpha peak, evoked potentials
**MRI**	
**Methods**	type (structural/functional), tesla, region of interest
**Analyses**	voxel-based morphometry, diffusion tensor imaging
