# Supplementary material for: Neurophysiological, balance and motion evidence in adolescent idiopathic scoliosis: A systematic review
Source: PLoS One. 2024 May 22;19(5):e0303086. doi: 10.1371/journal.pone.0303086 (PMC11111046; doi:10.1371/journal.pone.0303086)
Supplement: S3 Table — (PDF) [file pone.0303086.s003.pdf]

S3 Table

Quality assessment inventory.

**1. Aim of the work**
- Description of a specific, clearly stated purpose<br>- The research question is scientifically relevant
**2. Inclusion criteria (selection bias)**
- Description of inclusion and/or exclusion criteria
**3. Data collection & processing (performance bias)**
- Data collection is clearly described and reliable<br>- Data processing is clearly described and reliable<br>- Algorithms are clearly described and referenced
**4. Data loss (attrition bias)**
- Drop-outs $< 20\%$
**5. Statistical approach**
- Appropriate statistical analysis techniques<br>- Clearly states the statistical test used<br>- Actual probability values reported for the main outcomes
**6. Outcomes (detection bias)**
- Outcomes are topic relevant<br>- The work answers the scientific question stated in the aim
**7. Presentation of results**
- Presentation of the results is sufficient to assess the adequacy of the analysis<br>- The main findings are clearly described
